# Increased chromosomal stability in cultures of ovarian tumours of low malignant potential compared to cystadenomas

**DOI:** 10.1038/sj.bjc.6603817

**Published:** 2007-05-22

**Authors:** J Yu, D Roy, A D Brockmeyer, L Dubeau

**Affiliations:** 1 Department of Pathology, USC/Norris Comprehensive Cancer Center, Keck School of Medicine, University of Southern California, Los Angeles, CA 90089-9181, USA; 2 Department of Biochemistry, USC/Norris Comprehensive Cancer Center, Keck School of Medicine, University of Southern California, Los Angeles, CA 90089-9181, USA; 3 Department of Molecular Biology, USC/Norris Comprehensive Cancer Center, Keck School of Medicine, University of Southern California, Los Angeles, CA 90089-9181, USA; 4 Department of Obstetrics and Gynaecology, Division of Gynecologic Oncology, USC/Norris Comprehensive Cancer Center, Keck School of Medicine, University of Southern California, Los Angeles, CA 90089-9181, USA

**Keywords:** ovarian neoplasms, cellular aging, telomeres, aneuploidy, chromosomal stability

## Abstract

Cell cultures of ovarian cystadenomas transfected with SV40 large T antigen are not immortal because they invariably reach a phenomenon called crisis, which is triggered in part by telomere attrition. Recovery from crisis may be an integral component of the malignant transformation process. We reported earlier that such ovarian cystadenoma cell cultures undergo severe changes in DNA ploidy as they approach crisis and that such changes are an important determinant of crisis independent of telomere attrition. Here, we show that in sharp contrast to these benign ovarian tumours, the DNA content of ovarian tumours of low malignant potential (LMP) was remarkably stable as they approached crisis, suggesting that telomere attrition was the main determinant of this mortality checkpoint. Lack of a ploidy-based crisis was not due to loss of expression of a functional SV40 large T antigen protein. We conclude that ovarian LMP tumours are characterised by increased numerical chromosomal stability compared to cystadenomas. This might account for the fact that most LMP tumours are diploid or near diploid *in vivo*. This fundamental difference in chromosomal stability between ovarian cystadenomas and LMP tumours also suggests potential differences in predisposition to progression to malignancy between these two ovarian tumour subtypes.

The existence of a ‘semimalignant’ group of ovarian tumours was first described by Taylor (1929). It took an additional 40 years before such ovarian tumours, which are associated with a more favourable prognosis than their frankly malignant counterparts regardless of stage of presentation, became accepted as a clinical entity (Santesson and Kottmeier, 1968; Aure *et al*, 1971). Both the International Federation of Gynecology and Obstetrics (Serov *et al*, 1973) and the World Health Organization (Seidman *et al*, 2002) currently classify ovarian epithelial tumours as benign (cystadenomas), malignant (carcinomas), and low malignant potential (LMP, also called borderline). The latter is characterised by absent or minimal invasive potential, although they can spread outside the ovary and proliferate onto peritoneal surfaces. The exact nature and malignant potential of these tumours has remained controversial (Kurman and Trimble, 1993). They nevertheless represent a good model to investigate the molecular determinants of cancer development, because they show features that are intermediate between those of clearly benign and clearly malignant lesions.

Molecular genetic analyses performed over the last 15 years have identified several changes that distinguish ovarian LMP tumours from cystadenomas and carcinomas. For example, although somatic loss of heterozygosity, a hallmark of malignancy, has been occasionally observed in LMP tumours, it is clear that such events are not only rare in these tumours, but are also not essential to their development (Cheng *et al*, 1996). On the other hand, LMP tumours usually express telomerase (Wan *et al*, 1997), a feature of the malignant phenotype, and global DNA methylation changes (Cheng *et al*, 1997) or changes in the DNA methylation status of centromeric and juxtacentromeric sequences (Qu *et al*, 1999) in these tumours are more similar to those in carcinomas than in cystadenomas. Although these results strengthen the notion that LMP tumours are intermediate between benign and frankly malignant ovarian epithelial tumours, they shed little light on their underlying mechanisms. Mutations in specific protein kinases have been associated with LMP tumours and may be more frequent in these tumours than in carcinomas (Mok *et al*, 1993; Teneriello *et al*, 1993; Cheng *et al*, 2004; Ho *et al*, 2004). Such mutations are nevertheless seen in a large number of cancers of various types as well as in ovarian cystadenomas and their presence in LMP tumours sheds little light on the distinguishing molecular features responsible for the phenotypic differences between these tumours and either ovarian cystadenomas or carcinomas.

One of the most distinguishing characteristics of the malignant phenotype is the ability of cancer cells to proliferate indefinitely in tissue culture environments. Cultures derived from normal cells, in contrast, encounter mortality checkpoints after they have gone through a set number of mitoses. The first mortality checkpoint encountered by normal cells cultured *in vitro,* called senescence, is characterised by growth arrest due to inhibition of the cell cycle, whereas crisis, the next mortality checkpoint, is characterised by increased apoptosis (Hara *et al*, 1991; Shay *et al*, 1991). Although these phenomena have been observed primarily *in vitro*, it is likely that escape or recovery from both checkpoints is also important for the establishment of the malignant phenotype *in vivo*. We recently examined chromosomal changes associated with crisis in ovarian cystadenoma cells cultured *in vitro* (Velicescu *et al*, 2003). We used cells harbouring an expression vector for SV40 large T antigen and were therefore able to bypass senescence, as this antigen inhibits normal regulators of cell cycle activity. We showed that crisis was triggered by two independent phenomena that occurred sequentially in this cell culture system (Velicescu *et al*, 2003). The first was characterised by changes in DNA ploidy. Those changes were so severe that 10 population doublings after they became detectable by flow cytometry, the apoptotic pathways triggered by those genomic changes had resulted in rates of cell death that were higher than rates of cell proliferation. Cells that recovered from this ploidy-based crisis resumed their logarithmic growth, but eventually entered a second crisis state triggered by telomere attrition (Velicescu *et al*, 2003).

Here, we show that cultured LMP tumour cells did not undergo the severe ploidy changes that characterised cystadenomas as they aged *in vitro* and thus were spared from a ploidy-dependent crisis. Telomere attrition could be demonstrated in LMP tumours and thus was probably the main determinant of crisis in those cells in sharp contrast to cystadenomas. This genetic stability associated with cultured LMP tumours might account for the fact that such tumours tend to be diploid or near diploid *in vivo*. This fundamental difference between benign ovarian epithelial tumours and ovarian tumours of low malignant potential also suggests differences in predisposition to progression to malignancy between these two ovarian tumour subtypes.

## MATERIALS AND METHODS

### Cell lines and culture conditions

ML38 was derived from a mucinous LMP tumour. ML46 was derived from a serous LMP tumour with invasive implants. Both cell strains were isolated and infected with an adenovirus vector expressing SV40 large T antigen using published protocols (Luo *et al*, 1997). HOC-7 ovarian carcinoma cell line was obtained from Dr Ronald N Buick, University of Toronto (Buick *et al*, 1985). The source of ML10 was described earlier (Luo *et al*, 1997). ML38, ML46 and ML10 cell strains were grown in MEM supplemented with 10% FBS. HOC-7 cells were grown in RPMI supplemented with 10% FBS.

### Analysis of DNA ploidy by flow cytometry

One million cells were resuspended in phosphate-buffered saline (PBS) followed by fixation in 70% ethanol. The cell pellets obtained after centrifugation were resuspended in 1 ml of PBS, 10 *μ*g ml^−1^ propidium iodide and 100 *μ*g ml^−1^ RNase. The DNA contents of the cells were analysed using a Coulter Profile II flow cytometer (Beckman Coulter, Hialeah, FL, USA) and analysed using the MultiCycle software (Phoenix Flow Systems Inc., San Diego, CA, USA).

### Infection of cells with an adenoviral vector for SV40 large T antigen

We added 7.5 × 10^7^ PFU of an adenoviral vector expressing SV40 large T antigen to 90% confluent cultures of LMP cell lines in 35-mm tissue culture dishes. The cells were re-infected 48 h later. The source of the vector was described earlier (Luo *et al*, 1997).

### Western blot analysis

Cell monolayers were rinsed with 10 ml of ice-cold PBS and treated with ice-cold RIPA lysis buffer (20 mM Tris–HCl (pH 8.0), 125 mM NaCl, 0.5% NP-40, 20 mM NaF, 0.2 mM Na_3_PO_4_, 2 mM EDTA, 35 *μ*g ml^−1^ PMSF, 0.7 *μ*g ml^−1^ pepstatatin A, and 0.5 *μ*g ml^−1^ leupeptin) at 4°C for 30 min. They were detached from the tissue culture dishes (Becton Dickinson Labware, Franklin Lakes, NJ, USA) by rubbing with a cell lifter (Fisher Scientific, Pittsburgh, PA, USA). After centrifugation at 12 000 rpm for 30 min at 4°C, the supernatants were collected and stored at −80°C. Protein concentrations were determined using the BCA protein assay reagent kit (Pierce, Rockford, IL, USA). Samples containing 10 *μ*g of protein were electrophoresed on a 10% polyacrylamide gel and transferred onto nitrocellulose membranes (Biorad Laboratories, Richmond, CA, USA). The membrane was incubated overnight in 10% nonfat milk (Biorad Laboratories), 0.1% of PBS/Tween-20, and exposed to primary antibody for 1 h at room temperature. Following three 10 min washing in 0.1% PBS/Tween-20, the membrane was exposed to the secondary antibody coupled to horseradish peroxidase for 1 h at room temperature. The signal was detected by the ECL Western blotting detection reagents (Amersham Biosciences, Buckinghamshire, England). For loading control, the membrane was stripped in 100 mM *β*-mercaptoethanol, 2% SDS, 62.5 mM Tris–HCl (pH 6.7) at 65°C for 30 min, followed by extensive washing in 0.1% of PBS/Tween-20, and reprobed with monoclonal antibody against *β*-actin (Sigma, Saint Louis, MO, USA). The monoclonal antibody against SV40 large T antigen was obtained from Santa Cruz Biotechnology (Santa Cruz, CA, USA; cat no sc-147).

### Immunoprecipitation with p53 antibody

Two microgram of anti-p53 monoclonal antibody (Santa Cruz Biotechnology) was added to 500 *μ*g of total cellular protein extract (previously cleared by centrifugation) in a 1.5 ml microcentrifuge tube and incubated at 4°C for 2 h. Fifty microlitre of resuspended protein A-agarose was added and incubated at 4°C on a rocker overnight. Immunoprecipitates were collected by centrifugation at 2500 rpm for 5 min at 4°C. The supernatant was aspirated and the pellet was washed four times with 1.0 ml RIPA buffer (20 mM Tris–HCl (pH 8.0), 125 mM NaCl, 0.5% NP-40, 20 mM NaF, 0.2 mM Na_3_PO_4_, 2 mM EDTA, 35 *μ*g ml^−1^ PMSF, 0.7 *μ*g ml^−1^ pepstatatin A, and 0.5 *μ*g ml^−1^ leupeptin), resuspended in 20 *μ*l of 2 × electrophoresis sample buffer, and heated in boiling water for 2–3 min. After removing the agarose beads by centrifugation, 20 *μ*l aliquots of supernatant were electrophoresed on SDS-polyacrylamide gels followed by Western blotting with a monoclonal antibody against SV40 large T Antigen.

### Determination of telomere length by Southern blotting

Ten microgram of genomic DNA were digested with *Rsa*I/*Hin*fI restriction endonucleases, electrophoresed in 1% agarose gels, and transferred onto Zeta Probe GT membranes (BioRad Laboratories, Hercules, CA, USA) in 0.4 M NaOH following acid depurination in 0.4 N HCl. The probe, which consisted in a synthetic fragment containing three repeats of the human telomeric sequence TTAGGG_,_ was end labelled with ϒ-^32^P-dCTP. The membrane was hybridised in 0.5 M NaHPO_4_ (pH 7.2), 5% SDS, 1 mM EDTA (pH 8.0), 1% BSA, 50% formamide at 42°C overnight following pre-hybridisation for 1 h under the same conditions. The membrane was then washed for 15 min at 42°C in 2 × SSC, 1% SDS, followed by washing with 0.2 × SSC, 1% SDS for 15 min at room temperature. The hybridisation signals were visualised by exposure to a phosphoimager (Model GS-525, Molecular Imager® System, BioRad Laboratories).

### Detection of telomerase activity

Telomerase activity was measured using the Telomerase Repeat Amplification Protocol (TRAP) as described previously (Velicescu *et al*, 2003). Extracts from an immortal cell line (HOC-7) (Buick *et al*, 1985) were used as positive control.

## RESULTS

### Differences in ploidy stability between ovarian cystadenomas and LMP tumours expressing SV40 large T antigen

**Figure 1:**
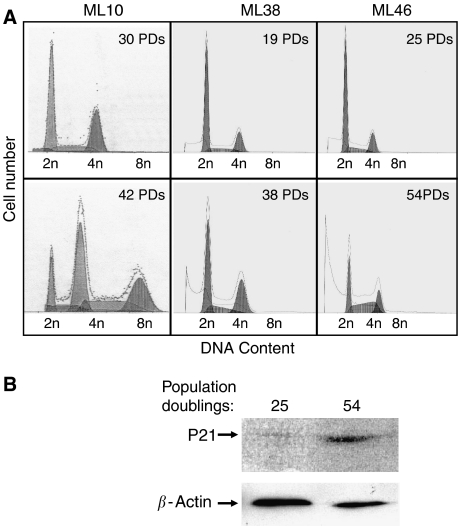
Influence of crisis on DNA content in ovarian cystadenoma *vs* LMP tumour cells cultured *in vitro*. *In vitro* cultures derived from either an ovarian cystadenoma (ML10) or from LMP tumours (ML38 and ML46) were harvested by trypsinisation, stained with propidium iodine, and analysed by flow cytometry at the indicated ages measured in population doublings (PDs) (**A**). In each case, the lower population doubling number represents cells growing logarithmically, whereas the higher population doubling number represents cells approaching crisis. Further evidence that the cells that reached the higher number of population doublings were in crisis can be obtained from the increased number of cells undergoing apoptosis seen on the DNA profiles in (**A**) or from demonstration of increased expression of p21 protein by Western blotting shown for ML46 in (**B**) and compared to expression of *β*-actin used as loading control.

Cultures of ovarian cystadenomas expressing SV40 large T antigen typically develop severe ploidy changes as they age in culture (Velicescu *et al*, 2003). We reported earlier that such changes in DNA content, and not telomere attrition, are primarily responsible for the initiation of crisis in these cells (Velicescu *et al*, 2003). We sought to determine whether ploidy changes also occurred in cultures derived from ovarian LMP tumours isolated using a similar protocol and harbouring the same SV40 large T antigen expression vector. Three cell cultures, one derived from an ovarian cystadenoma called ML10 and two derived from LMP tumours, respectively, called ML38 and ML46, were analysed for cellular DNA content by flow cytometry either during their logarithmic growth phase (Figure 1A, top panels) or as they approached crisis (Figure 1A, bottom panels). This pre-crisis period in each culture was determined based on reduced average population doubling rates and on the presence of an increasing number of swollen or floating cells in the culture dishes. Although most of the ML10 cells were near tetraploid, as they approached crisis in agreement with our earlier report based on observations of three different cystadenoma cell strains (Velicescu *et al*, 2003), the two cultures derived from LMP tumours showed little changes in their DNA content throughout their crisis phase. The near diploid status of ML38 and ML46 cells and the near tetraploid status of ML10 cells approaching crisis was also confirmed by counting the number of metaphase chromosomes in each cell line (data not shown). Further evidence that the ML38 and ML46 cells shown in Figure 1 had indeed entered a crisis phase after 38 and 54 population doublings comes from the increased number of cells with a DNA content less than 2n, a sign of apoptosis, seen in the respective panels (Figure 1A) as well as from the demonstration of increased expression levels of p21, another marker of crisis, as shown in Figure 1B for ML46.

### Absence of ploidy changes in LMP tumours approaching crisis is not due to loss of a functional SV40 large T antigen

**Figure 2:**
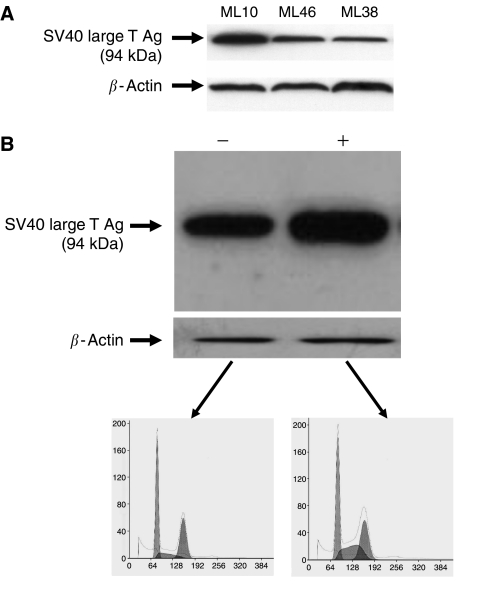
SV40 large T antigen expression in LMP tumour cells. (**A**) Protein extracts were obtained from ML10, ML38, and ML46 cells and analysed by Western blotting using antibodies against either SV40 large T antigen or *β*-actin. (**B**) ML46 cells that had undergone 44 population doublings in culture were either not re-infected (−) or re-infected (+) with the adenoviral vector for SV40 large T antigen and analysed as they approached crisis 10 population doublings later by Western blotting with the same antibodies and by flow cytometry following staining with propidium iodide.

It is well known that cells expressing SV40 large T antigen typically develop changes in their DNA ploidy similar to those observed in ovarian cystadenoma cell strains such as ML10. We therefore considered the possibility that the notable lack of ploidy alterations in cultures of LMP tumours could be due to either loss of the SV40 large T antigen vector or to silencing of this vector from either mutation or DNA methylation changes. We compared the levels of this antigen in ML10, ML38, and ML46 cells by Western blot analysis. The results showed expression of this protein in all cell lines (Figure 2A), ruling out the possibility of complete loss of the vector in the cells derived from LMP tumours. We re-infected ML46 cells with our adenovirus vector expressing SV40 large T antigen, because we noted that the amounts of T antigen protein present in both LMP tumour cell strains were lower than in ML10. Although this resulted in a substantial increase in the levels of intracellular T antigen protein that was still apparent 10 population doublings later (Figure 2B), there were no significant differences in DNA content between cells not subjected *vs* subjected to re-infection with the adenoviral vector at this time point (Figure 2B). We conclude that reduced SV40 large T antigen expression in ML38 and ML46 did not account for the relative stability of their DNA content compared to ML10 cells. The reason for the lower total levels of this antigen in ML38 and ML46 cells when compared to ML10 may, in part, be due to the increased gene dosage in ML10 due to increased DNA ploidy in the pre-crisis period.

**Figure 3:**
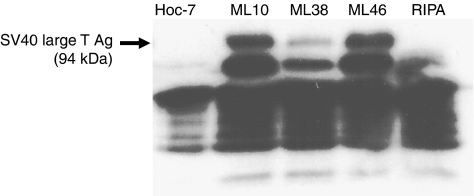
SV40 large T antigen expressed in LMP tumour cells is functional. Protein extracts from HOC-7 ovarian carcinoma cells, ML10, ML38, and ML46 cells were immunoprecipitated with a monoclonal antibody against p53 and analysed by Western blotting using a monoclonal antibody against SV40 large T antigen. As control, immunoprecipitation with anti-p53 was performed on a buffer sample containing no protein extract (RIPA).

The possibility remained that the SV40 large T antigen expressed in the LMP cell lines had reduced activity due to the presence of a mutation. We therefore determined whether the T antigen protein expressed in both cell lines had retained its ability to bind to p53. Binding to this protein is an important determinant of the consequences of T antigen expression on cell cycle activity. Whole-cell extracts of ML38 and ML46 were immunoprecipitated with anti-p53 antibody and the resulting precipitates were analysed by Western blotting using an anti-large T antigen antibody. The results (Figure 3) confirmed that the T antigen protein had coprecipitated with p53 in ML10, ML38, and ML46 cells. There was no detectable level of T antigen in HOC-7 ovarian carcinoma cells, which were used as control. Although the levels of T antigen that coprecipitated with p53 in the experiment shown in Figure 3 appeared lower in ML38 cells than in ML10 cystadenoma cells, coprecipitation nevertheless did occur in those cells and the levels in ML46 were similar to those present in ML10. We conclude that the relative ploidy stability of the LMP cell lines is not due to absence of a functional SV40 large T antigen, at least based on ability of this antigen to bind to p53.

### Telomere length in cultured LMP cells

**Figure 4:**
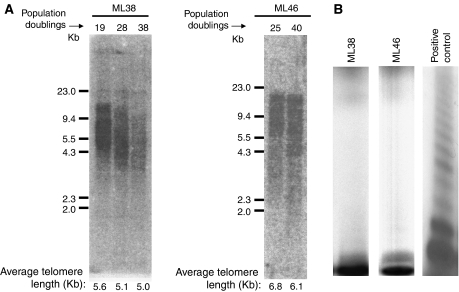
LMP tumour cells undergo telomere attrition and lack telomerase activity. (**A**) Genomic DNA was extracted either from ML38 or ML46 cells after they reached the indicated number of population doublings and analysed by Southern blotting using a radiolabelled probe complementary to the human telomeric sequence. The figure shows an autoradiograph of the Southern blots after hybridisation with this probe. Average telomere lengths were calculated by integrating the intensity of the signal corresponding to various sizes with the help of a phosphoimager. (**B**) Protein extracts from ML38 and ML46 were assayed for telomerase activity using the TRAP assay protocol. The radioactive products were electrophoresed on 8% polyacrylamide and visualised by autoradiography. Small quantities of a protein extract from HOC-7 cells, which are known to express telomerase, were added to extract from ML46 and used as positive control. Lack of the characteristic ladder in ML38 and ML46 indicates absence of telomerase activity (Counter *et al*, 1992).

We previously showed that telomere attrition is not the main determinant of crisis in cultured ovarian cystadenoma cells, because these cells characteristically show telomeres of a length sufficient to support further chromosome replication by the time they reach crisis (Velicescu *et al*, 2003). It is the severe ploidy changes that invariably accumulate in these cells that trigger the phenotypic manifestations of crisis, in part via induction of p21 resulting in increased apoptosis (Velicescu *et al*, 2003). Our earlier study further showed that telomere attrition only became significant in cystadenoma cells that had recovered from this ploidy-based crisis (Velicescu *et al*, 2003). It is likely that, unlike in cystadenomas, crisis is predominantly determined by telomere attrition in LMP tumours, because DNA content in those cells is more stable, depriving them of a stimulus for a ploidy-based crisis. We performed Southern blotting analyses of genomic DNA extracted from ML38 and ML46 cells of different ages to examine the extent of telomere attrition in those cells. As expected, there was a progressive shortening of telomere length seen in ML38 cells at the various time points examined (Figure 4). In addition, although sufficient quantities of ML46 DNA could not be obtained at 54 population doublings, because of extensive apoptosis, telomere attrition was already evident in those cells at 40 population doublings (Figure 4A). In addition, none of the two LMP cell cultures expressed telomerase activity detectable by the TRAP assay protocol (Figure 4B). Taken together, these results suggest that cultured LMP cells do not have the ability to maintain telomere length and presumably undergo a telomere-driven crisis.

## DISCUSSION

Our results clearly show that cultures of ovarian LMP tumour cells expressing SV40 large T antigen remained diploid and showed remarkable stability in their ploidy status throughout their *in vitro* life span. This was in sharp contrast to cultures of ovarian cystadenomas expressing the same antigen and isolated using similar protocols, which typically become polyploid and eventually aneuploid as they approach the phenomenon of *in vitro* crisis as demonstrated in a previous report (Velicescu *et al*, 2003) and illustrated again in this paper using a representative cystadenoma cell strain. These differences were not due to loss of a functional SV40 large T antigen in the cultures of LMP tumours. Given that only two LMP tumour cell lines were available for these studies, the generality of those differences between cystadenomas and LMP tumours has not been established. However, if verified in a larger population of LMP tumours, these results point to a fundamental difference between ovarian cystadenomas and LMP tumours, with important implications on the genetic stability of these ovarian tumour subtypes.

Most LMP tumours express telomerase *in vivo*, providing an additional argument in support of the notion that they represent a stage of malignant transformation that is distinct from cystadenomas (Wan *et al*, 1997). Given that telomere attrition is thought to be associated with structural chromosomal instability that can be overcome by telomerase (Counter *et al*, 1992; Artandi *et al*, 2000; O’Hagan *et al*, 2002), one may question whether the increased stability observed in our cultured LMP tumour cells could have been due to varying levels of telomerase expression. However, LMP tumour cell lines do not maintain telomerase expression once put in culture. We not only have been unable to detect telomerase activity in cultured LMP tumour cells using the TRAP assay, but also the cells underwent telomere attrition culminating in crisis. Thus, the mechanism responsible for the increased stability seen in cultured LMP tumour cells appears to be independent of telomerase activity. A better understanding of the mechanisms underlying the apparent intrinsic genomic stability associated with LMP tumours might provide insights into molecular markers that could potentially help in the histopathological diagnosis of LMP tumours.

Acquisition of replicative immortality is an essential component of the malignant phenotype. It is likely that the phenomena of senescence and crisis are not mere *in vitro* curiosities, but also have *in vivo* counterparts that need to be overcome during the process of cancer development. Our results suggest that LMP tumours, but not cystadenomas, may have developed a mechanism that protects them against numerical chromosomal instability, allowing these tumours to overcome at least one of the road blocks to replicative immortality that operates in cystadenomas. This might account for the fact that the large majority of these tumours are diploid or nearly diploid *in vivo*, in spite of their rapid proliferation rate (Friedlander *et al*, 1984; Lai *et al*, 1996). In fact, aneuploid LMP tumours are associated with a more aggressive clinical course (Kaern *et al*, 1990; Lai *et al*, 1996; Sykes *et al*, 1997) and their response to chemotherapeutic agents may be more typical of ovarian carcinomas (Kaern *et al*, 1990; Burger *et al*, 2000), raising the possibility that at least some of the those tumours are carcinomas incorrectly diagnosed as LMP tumours. Indeed, the possibility of using ploidy status as a diagnostic tool to help distinguishing ovarian LMP tumours from carcinomas has been suggested (Friedlander *et al*, 1984).

We reported earlier that, although p53 mutations are rare in solitary cystadenomas, such mutations, when present, are concordant in both components of heterogeneous tumours characterised by a clearly malignant portion contiguous to a morphologically benign lesion (Zheng *et al*, 1995). This, as well as other observations (Zheng *et al*, 1993), led us to conclude that ovarian cystadenomas are unlikely to progress spontaneously to carcinoma unless predisposing changes such as a p53 mutation are present. The cell culture system used in our study, which is based on cells expressing SV40 large T antigen, is relevant to this scenario because this antigen is known to inactivate p53, resulting in the equivalent of a p53 mutation. The fact that cystadenomas expressing SV40 large T antigen are prone to numerical chromosomal instability, whereas LMP tumours expressing the same antigen are stable with regard to their ploidy status suggests that specific genetic alterations such as p53 mutations are more likely to lead to progression to a malignant lesion when they occur in cystadenomas compared to LMP tumours. Moreover, if an LMP tumour does progress to malignancy, the resulting carcinoma might be more likely to remain diploid or near diploid, implying a lower histological grade. This idea supports the conclusion of Shih and Kurman, who suggested that low-grade ovarian serous carcinomas, but not high-grade carcinomas, may develop in pre-existing LMP tumours (Shih and Kurman, 2005).
